# RGB-Depth Camera-Based Assessment of Motor Capacity: Normative Data for Six Standardized Motor Tasks

**DOI:** 10.3390/ijerph192416989

**Published:** 2022-12-17

**Authors:** Hanna Marie Röhling, Karen Otte, Sophia Rekers, Carsten Finke, Rebekka Rust, Eva-Maria Dorsch, Behnoush Behnia, Friedemann Paul, Tanja Schmitz-Hübsch

**Affiliations:** 1 Experimental and Clinical Research Center, a Cooperation between the Max-Delbrück-Center for Molecular Medicine in the Helmholtz Association and the Charité—Universitätsmedizin Berlin, 13125 Berlin, Germany; 2 Experimental and Clinical Research Center, Charité—Universitätsmedizin Berlin, Corporate Member of Freie Universität Berlin and Humboldt-Universität zu Berlin, 13125 Berlin, Germany; 3 Max-Delbrück-Center for Molecular Medicine in the Helmholtz Association (MDC), 13125 Berlin, Germany; 4 Motognosis GmbH, 10119 Berlin, Germany; 5 Department of Neurology, Charité—Universitätsmedizin Berlin, Corporate Member of Freie Universität Berlin and Humboldt Universität zu Berlin, 10117 Berlin, Germany; 6 Berlin School of Mind and Brain, Humboldt-Universität zu Berlin, 10117 Berlin, Germany; 7 NeuroCure Clinical Research Center, Charité—Universitätsmedizin Berlin, Corporate Member of Freie Universität Berlin and Humboldt Universität zu Berlin, 10117 Berlin, Germany; 8 Department of Psychiatry and Psychotherapy, Charité—Universitätsmedizin Berlin, Corporate Member of Freie Universität Berlin and Humboldt Universität zu Berlin, 12203 Berlin, Germany

**Keywords:** instrumental motion analysis, normative data, RGB-Depth camera, Microsoft Kinect v2, gait analysis, tandem gait, postural control, stepping in place, standing up and sitting down

## Abstract

Background: Instrumental motion analysis constitutes a promising development in the assessment of motor function in clinical populations affected by movement disorders. To foster implementation and facilitate interpretation of respective outcomes, we aimed to establish normative data of healthy subjects for a markerless RGB-Depth camera-based motion analysis system and to illustrate their use. Methods: We recorded 133 healthy adults (56% female) aged 20 to 60 years with an RGB-Depth camera-based motion analysis system. Forty-three spatiotemporal parameters were extracted from six short, standardized motor tasks—including three gait tasks, stepping in place, standing-up and sitting down, and a postural control task. Associations with confounding factors, height, weight, age, and sex were modelled using a predictive linear regression approach. A z-score normalization approach was provided to improve usability of the data. Results: We reported descriptive statistics for each spatiotemporal parameter (mean, standard deviation, coefficient of variation, quartiles). Robust confounding associations emerged for step length and step width in comfortable speed gait only. Accessible normative data usage was lastly exemplified with recordings from one randomly selected individual with multiple sclerosis. Conclusion: We provided normative data for an RGB depth camera-based motion analysis system covering broad aspects of motor capacity.

## 1. Introduction

Identification and monitoring of motor impairments are key elements in the management of diseases impacting motor function. The instrumental task-based assessment of motor capacity provides an alternative to observation and assessment by clinical experts and analog standardized tests, such as the Timed Up and Go Test [1] or Timed 25-Foot Walk [2]. Due to anticipated time and cost efficiency as well as outcome objectivity, instrumental motion analysis has drawn increasing attention in recent years. Telemedical use of such technologies from patients’ homes can further protect vulnerable groups and provide relief for overburdened healthcare systems.

Interpretability of outcomes from instrumental motion analysis is, however, still limited. Heterogeneous usage of different technologies, movement protocols, extracted parameters and respective algorithms rarely allows for robust between-system compatibility. Reliable normative data for the system in use thus comprises a crucial prerequisite for the interpretation of outcomes. Respective values can help to define meaningful thresholds for assumed pathology, expected variability, as well as dependencies on anthropometric and demographic features. Normative datasets can further aid in improving harmonization by revealing systematic biases between system outputs.

Large normative datasets of gait parameters ranging from gait speed to arm swing asymmetry continue to be of high interest to the scientific community and are comparatively prevalent [3,4,5,6,7]. Few larger databases exist for other motor tasks, such as tandem gait and postural control [8,9,10]. However, most reported parameter values from healthy controls stem from small case-control or proof-of-concept studies and comprise around 30 healthy subjects or less [11,12,13,14,15,16,17,18,19,20,21]. For this study, a motion analysis system based on the use of a single RGB-Depth camera (Microsoft Kinect v2) was employed, which has been evaluated for accuracy and reliability [11,22] and has been used in various clinical populations [5,23,24]. Previous related works from our groups include outcome parameters from healthy controls [23,25,26,27,28,29], but these do not represent a robust normative database by themselves due to likewise limited sample sizes, restriction to single motor tasks, or general study design.

In this study, we thus aimed to provide elaborate normative values for spatiotemporal parameters of an RGB-Depth camera-based motion analysis system for six different motor tasks. We further assessed associations of the parameters with confounding demographic and anthropometric factors and illustrated usage of the normative data.

## 2. Materials and Methods

### 2.1. Participants

One-hundred-thirty-three participants were pooled from control groups of two multiple sclerosis studies (acronyms: Valkinect, VIMS) and one autism spectrum disorder study (acronym ASD) at Charité—Universitätsmedizin Berlin, Berlin, Germany. Participants were recruited via social media posts, institutional databases, intranet, and by approaching accompanying persons from respective case cohorts.

Exclusion criteria were psychiatric disorders, chronic neurological diseases, or acute motor impairments. Adapted from norm data specifications for another commercially available system [30,31], we used five persons per sex and age decade as a lower limit in sample size planning. We focused on adult, decidedly non-geriatric, individuals and thus included participants within the ages of 20 and 60, representing an adequate control group for common neuroimmunological conditions. All included participants received instrumental motion analysis and had complete information regarding age, sex, height, and weight (Table 1, visualized in Supplementary Figure S1).

### 2.2. Instrumental Motion Analysis

**Figure 1:**
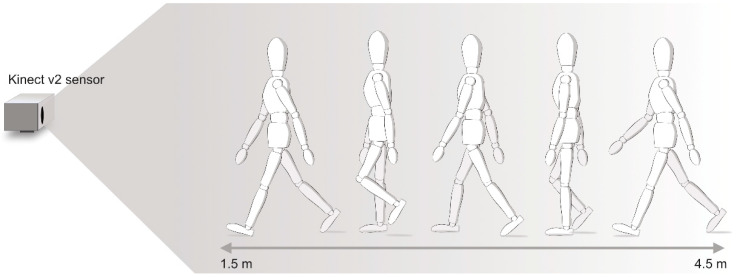
Technical set-up of the Motognosis Labs motion analysis system with a single Microsoft Kinect v2 sensor with exemplary sketch of a gait task. The illustration was included by courtesy of Motognosis GmbH.

Labs (Motognosis GmbH, Berlin, Germany; versions 1.4.0.2, 1.4.0.3, 2.0.1.0, 2.1.2.0), and a markerless motion analysis system based on a single RGB-Depth consumer camera (Microsoft Kinect v2; Microsoft cooperation, Redmond, WA, USA), were used to record short movement tasks from participants wearing standard clothing and comfortable footwear in a 1.5–4.5 m distance from the sensor (Figure 1). The Kinect v2 sensor was positioned at a height of 1.4 m and tilted in a pitch direction of roughly −8° to −9°. Scientific staff operated the system following written standard operating procedures for technical setup and task instruction.

We included data for six tasks: short comfortable speed walk (SCSW), short maximum speed walk (SMSW; VIMS and Valkinect only as this task was not included in the ASD measurement protocol), tandem gait referred to as short line walk (SLW), stepping in place (SIP), standing up and sitting down (SAS; VIMS and Valkinect only as this task was not included in the ASD measurement protocol), and postural control (POCO). Within each session SCSW, SMSW, SLW, and SAS were recorded three consecutive times, SIP and POCO were recorded once.

The Microsoft Kinect SDK (version 2.0.14) enables extraction of 25 three-dimensional time series of body landmarks from these recordings, which were used to extract spatiotemporal parameters with custom algorithms. Here, we extracted 43 parameters (Table 2), most of which have been previously introduced [22,23,25,26,27,28]. Others were carefully vetted in terms of clinical interest and statistical properties when tested in an independent dataset [22].

### 2.3. Data Analysis

Recordings with gross performance deviations (e.g., wrong feet position for POCO) or technical errors were identified using a previously described post hoc quality control pipeline [33] and discarded. Statistical analyses were performed, and visualizations were generated using Python 3.7.3 (packages pandas 1.3.5, numpy 1.21.6, statsmodels 0.13.2, seaborn 0.11.2, matplotlib 3.1.0, scipy 1.7.3, scikit-learn 0.21.2). For SCSW, SMSW, SLW and SAS, the extracted spatiotemporal parameters were averaged per participant over all remaining repetitions. Data are presented as group mean, standard deviation, coefficient of variation, and quartiles and distributions are visualized.

To model and address the influence of potential confounders in a generalizable way, we fitted ordinary least squares regression models—*parameter ~ age + sex + height + weight + study*—where sex was dummy-coded (female: 0; male: 1), and the study was used as an effect-coded control variable. Models were first fitted in a repeated (100 times) five-fold cross-validation procedure, using the $R^{2}$-value ($R_{test}^{2}$) between true and predicted parameter values of respective test sets at each fold, and repetition as a performance indicator. Predicted parameter values were calculated using derived *β*-values from respective training sets, omitting the study to simulate assessing model performance on external data. For an averaged $R_{test}^{2}$ > 0.1, models were assumed to describe generalizable associations. For these models *β*-values, corresponding 95% confidence intervals and *p*-values for the independent variables were extracted after fitting them on the full dataset.

For supplementary information, we extracted bivariate statistics (Pearson’s correlation coefficient, independent samples *t*-test, one-way ANOVA) regarding associations between spatiotemporal parameters and potential confounders.

To better interpret individual behavior in comparison to normative data and across different parameter scales, we applied z-score normalization. The z-score of a value refers to its relative distance to the mean measured in numbers of standard deviations:

(1) $$z_{raw,\;i}=\frac{x_{i}-\bar{x}\;}{s},$$

with $x_{i}$ being the raw value for a given parameter and subject *i*, and $\bar{x}$ and *s* being the raw normative data’s sample mean and standard deviation. For spatiotemporal parameters with previously detected confounding associations with age, sex, height and weight, respectively standardized residuals of the linear models were favored over $z_{raw}$:

(2) $$z_{res,i}=\frac{\varepsilon_{i}}{s_{\varepsilon }},\;$$

with $s_{\varepsilon }$ being the standard deviation of the normative residuals and

(3) $$\varepsilon_{i}=x_{i}-(\beta_{0}+\beta_{Age}x_{i,Age}+\beta_{Sex}x_{i,Sex}+\beta_{Height}x_{i,Height}+\beta_{Weight}x_{i,Weight}).$$

## 3. Results

### 3.1. Normative Values

**Figure 2:**
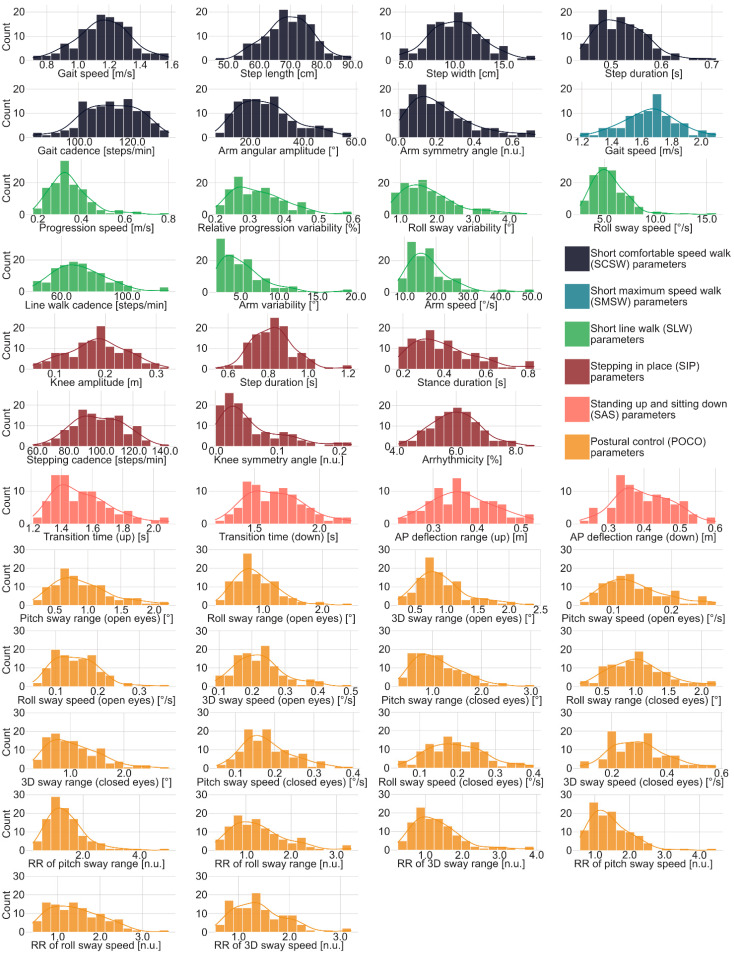
Distributions of raw spatiotemporal parameters color coded by motor task. Abbreviations: AP: anterior-posterior; n.u.: unitless; POCO: postural control; RR: Romberg ratio; SAS: standing up and sitting down; SCSW: short comfortable speed walk; SIP: stepping in place; SLW: short line walk; SMSW: short maximum speed walk.

Distributions and statistics presented in this section were produced using only data that passed quality control, performed by one trained researcher. Discard rates (3.2–15.0% depending on task) and reasons for exclusion are provided in supplementary Table S1. The normative values are distributed highly variable (Figure 2): SCSW gait speed, for instance, is approximately normally distributed, while other parameters such as SLW roll sway speed are highly skewed or feature outliers. Respective descriptive statistics are provided in Table 3.

### 3.2. Associations with Age, Sex, Height, and Weight

Negative and low mean $R_{test}^{2}$ values (Table 3) indicated that most fitted models did not generalize well when presented with the new data and modelled confounding associations were not sustainable for this dataset. $R_{test}^{2}$ values greater than 0.1 were only observed for SCSW step length and step width. For these parameters, linear models fitted using the full dataset showed an association of increased step length in taller, lighter individuals and increased step width in heavier, male individuals (Table 4). We thus suggest normalizing new datapoints for these spatiotemporal parameters using the provided models (Table 4) and (3). Resulting residuals should then be compared to residuals of the normative data using (2). Bivariate statistics regarding associations between spatiotemporal factors age, sex, height, weight, and study are provided in Supplementary Table S2.

### 3.3. Usage of Normative Values

**Figure 3:**
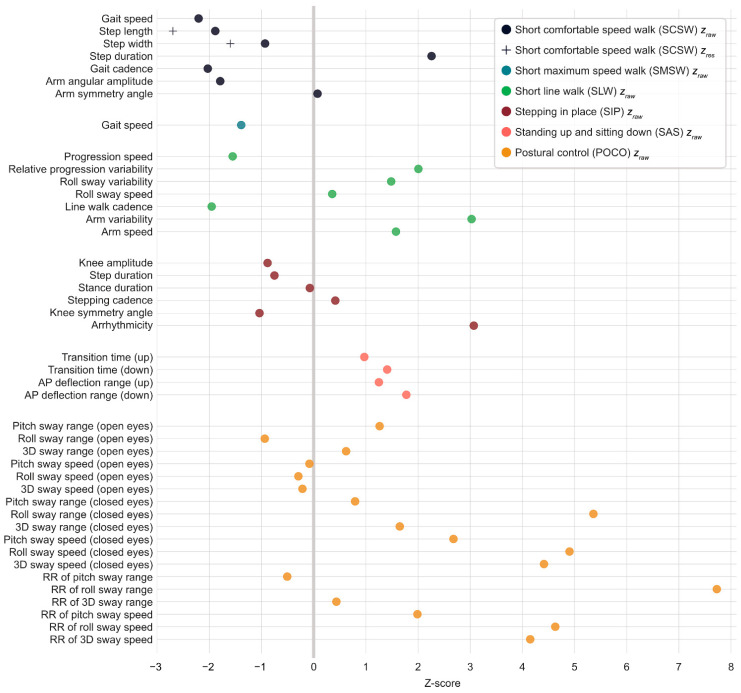
Z-score transformed spatiotemporal parameters from recordings of a randomly picked person with multiple sclerosis from the Valkinect study. Abbreviations: AP: anterior-posterior; RR: Romberg ratio; *z_raw_*: z-score for raw values; *z_res_*: z-score for residual values.

Usage of the provided normative data was exemplified for data from a person with multiple sclerosis (Figure 3). The illustration of z-score transformed values allows for straightforward overview of individual patterns, and cross-checking whether findings from the literature apply for the individual at hand. For instance, the person in Figure 3 shows above (healthy) average POCO pitch/roll/3D sway speed with closed eyes and below average SMSW gait speed, which has been likewise found at group level in pilot studies using Motognosis Labs and the Kinect v1 (healthy participant overlap with Valkinect and VIMS: n = 9) [26,27].

For SCSW step length and step width, we suggest using *z_res_* over *z_raw_* in visualizations and further analysis. In Figure 3, both are depicted to illustrate effects of regression-based normalization for identified confounding associations. The patient shows below average SCSW step length and step width when looking at raw data (54.86 cm and 7.65 cm; *z_raw_* = −1.88 and −0.93). These values deviate further from the healthy mean when controlling for age, sex, height, and weight (*z_res_* = −2.70 and −1.60).

## 4. Discussion

In this study, we provided normative data for 43 spatiotemporal parameters of six short motor tasks recorded from 20- to 60-year-old healthy adults using an inexpensive and easy-to-use RGB depth camera system. The necessity for regression-based normalization regarding demographic and anthropometric confounders was found mostly negligible for this sample except for two gait parameters. Further use of the raw and normalized normative parameters was exemplified by means of z-score visualizations.

The presented values will aid interpretation of outcomes for clinical users and researchers employing instrumental motion analysis—especially RGB-Depth camera systems. At group level, the results can serve for hypothesis generation about populations of interest, even without a sufficiently matched control group. Observed patterns may define univariate or multivariate “motor biomarkers” indicating pathology. For longitudinal studies, cross-sectional normative data is arguably less relevant, as subjects provide their own baseline data. However, it can still aid overall interpretation of intraindividual changes. For instance, changes of a similar magnitude might be clinically more meaningful if they exceed certain normative thresholds.

### 4.1. Short Comfortable and Maximum Speed Walk (SCSW and SMSW)

Gait speed measurement highly depends on start protocol (static or dynamic), path length, and speed instructions. Our means (SCSW: 1.16 m/s; SMSW: 1.66 m/s) are consistent with values for adults under 60 recorded with a stopwatch (4 m gait, static start; SCSW: 1.11–1.21 m/s; SMSW: 1.57–1.88 m/s) [3] and a Kinect v2 study by Latorre et al. (measurements starting at 6 m from the sensor; SCSW: 1.16–1.19 m/s) [5]. However, SMSW gait speed in Motognosis Labs studies with the Kinect v1 (healthy participant overlap with Valkinect and VIMS: n = 9) substantially exceeded our results (1.83 and 1.85 m/s) [27,28], likely because of a more dynamic starting protocol. The limited sensor range of the Kinect v2 only allows for few SMSW gait cycles to be recorded. Thus, parameters other than speed lack robustness [33] and were not reported here.

Latorre et al. further report comparable SCSW cadence (107.43–112.37 steps/min versus our 112.07 steps/min), but divergent step lengths and widths (62–67 cm and 11–12 cm versus our 69.35 cm and 10.19 cm) [5], which may result from a differing set-up and algorithmic step definition.

In line with our findings, mean arm angular amplitudes of 25.0–26.2° were measured during 4 km/h treadmill walking with an ultrasound motion capture system [7]. During 1-min walking at preferred speed in adults younger than 60, Mirelman et al. measured considerably higher arm swing amplitudes (42.0–53.4°) but lower asymmetry (corresponding to an arm symmetry angle of 0.164–0.202) [6]. A comparison with other asymmetry data from the literature was mostly inconclusive because of different metrics, e.g., in [7].

Despite clinically well-established effects, age did not emerge as a relevant confounder for gait parameters—possibly because of our comparatively young cohort. This is consistent with findings that gait speed does not change significantly under the age of 60 [3,4]. We expected to find associations with height, as respective gait parameter normalization approaches have long been proposed and comprise for example scaling as a function of leg length [34] or body height [35]. The extent to which size differences explain sex or weight differences and vice versa cannot be reliably determined using our statistical approach.

### 4.2. Short Line Walk (SLW)

Test performance of SLW can reveal subtle balance deficits and depends highly on instructions and individual implementation strategies. Conventionally reported SLW parameters include time-to-complete (stopwatch measure) and number of missteps (observational measure) [36], only few studies regarding instrumental motion analysis of SLW are available.

Velázquez-Pérez et al. derived trunk, lumbar and arm ranges of motion using wearables, which are kindred parameters to SLW arm or roll sway variability and speed, but not directly comparable [37]. Grinberg et al. focused on lower limb gait parameters during 3 m tandem gait at self-selected speed and found a substantially higher line walk cadence compared to our results (84.5 steps/min vs. 71.78 steps/min) [21]. Ganz et al., on the other hand, provide rather synoptic parameters derived from a single wearable that describe postural corrections, overall movement, as well as regularity and complexity. They reported composite factors of these parameters to be associated with age, sex, and BMI in older adults [8]. However, time-to-complete was reported not to be associated with age, sex, or BMI [36], which is more in line with our results that yielded no robust model describing associations between spatiotemporal parameters and respective confounders.

### 4.3. Stepping in Place (SIP)

The observed mean stepping cadence of 98.29 steps/min compared well to the 99 steps/min reported by Garcia et al. Interestingly, they found similar cadences for SIP and SCSW in their samples [14], while SIP stepping cadence was substantially lower than SCSW gait cadence here. Other authors implicitly report higher mean cadence (104.35–112.15 steps/min; extrapolated from reported cycle durations) and lower arrhythmicity (2.63–3.89) [13,15]. Substantially higher arrhythmicity (slightly adapted algorithm) was measured in persons with Parkinson’s disease using Motognosis Labs [23], yielding the parameter as a potential gait variability substitute. In [23] further parameters, such as longest stance time, were extracted to assess festination or freezing of gait behavior. This was omitted here, as no such behavior was expected in healthy adults.

### 4.4. Standing up and Sitting down (SAS)

Although sit-to-stand transitions are widely used in clinical ratings or timed assessments of various disorders (e.g., as part of the Timed Up and Go Test), heterogeneous transition phase definitions, phase segmentation procedures and outcome parameters obstruct direct comparisons to our data.

For instance, Weiss et al. [17,18] reported comparatively short mean durations for Sit-to-Stand (0.5 s and 0.56 s) and Stand-to-Sit transitions (0.7 s and 0.85 s) during performance of the Timed Up and Go Test.

However, they took the extrema of accelerometer-derived anterior-posterior acceleration for phase segmentation, which systematically underestimates these phases, when considering respective formal definitions [19]. Definitions from van Lummel et al. are more consistent with our approach and yielded slightly lower values (1.45 s and 1.47 s) during five times Sit-To-Stand at self-selected speed in young adults [16].

While we found low inter-individual variability and negligible confounding for transition times in our sample, differences in transition times were previously described between age-groups 25 and younger and 70 and older [16,20]. Furthermore, possible cultural bias has been observed for this task [25].

### 4.5. Postural Control (POCO)

Direct comparison to data from the literature is futile due to major differences in measurement technologies (e.g., force plates, pressure plates, and accelerometers), motor tasks (e.g., reaching, single-legged, and open stance tasks) and outcome measures (e.g., path lengths and displacement of center of pressure) [9,10,11,12].

Previously published values from healthy adults using Motognosis Labs with a Microsoft Kinect v1 (healthy participant overlap with Valkinect and VIMS: n = 9) feature slightly lower sway speed values and slightly higher respective Romberg ratios. They propose using the 95th percentile of 3D sway speed (closed eyes) in healthy controls as a threshold for abnormal sway in persons with multiple sclerosis, which amounts to 0.50°/s and compares well to our data (95th percentile = 0.47°/s; not explicitly reported here). Consistent with our findings, they reported no associations with age, sex, height, or BMI [26]. In studies with a broader age range, increased center of pressure sway paths in balance tasks were, however, reported to be associated with older age and male sex [9,10].

### 4.6. Z-Score Transformation and Visualization

Comprehensible data visualization greatly increases interpretability and usability of outcomes for experienced and technology-naïve users alike. Z-score transformations are well established considering, e.g., neuropsychological testing [38], and relate to visualizations used in the usual lab report format, which is highly familiar to clinical users. In instrumental motion analysis, such transformations and visualizations are used less frequently. Notably, however, z-scores are visualized alongside metric value and percentile representations for the commercially available Mobility Lab v2 system (APDM Inc., Portland, OR, USA) [30].

### 4.7. Limitations

Our sample is biased demographically towards a German, Caucasian, and urban population, which potentially influences motor behavior [25,39,40]. However, such biases can be counteracted by comparing our results with databases from other study sites, social or cultural groups [25].

Expanding the data set may generally lead to more stable estimates of normative values and associations with confounders. For expansion, more demographically and anthropometrically extreme data should be used where appropriate, e.g., from older subjects when investigating neurodegenerative diseases such as Parkinson’s disease.

In terms of analysis, we restricted our modelling of confounding effects to linear associations. Further, advantages of z-scores are limited for non-normally distributed parameters, e.g., direct conversion into percentiles is not possible. They still serve the general purpose of normalization, but, depending on use case, other transformations could be explored. Lastly, the participant used for exemplification of the z-score visualizations was chosen at random and does not necessarily show representative motor behavior.

## 5. Conclusions

The reported normative values fill existing gaps in the literature of motion capture for various tasks assessing motor capacity as well as generally RGB-Depth camera-based motion analysis. The results will inform clinicians and researchers on how to effectively use and interpret the outcomes of this technology.

## Figures and Tables

**Table 1:** Anthropometric and demographic subject characteristics overall and subdivided by study. Abbreviations: SD: standard deviation.

Study	Sample Size (% Female)	Age Mean (SD; Range) [Years]	Height Mean (SD; Range) [cm]	Weight Mean (SD; Range) [kg]	BMI Mean (SD; Range) [kg/m2]
All	133 (56%)	36.83 (10.44; 20–60)	172.89 (9.34; 153–194)	71.80 (13.86; 46–115)	23.94 (3.79; 17.75–34.33)
ASD	41 (51%)	33.88 (7.99; 20–49)	174.17 (9.65; 155–194)	73.85 (16.06; 46–115)	24.24 (4.41; 17.75–33.90)
VIMS	57 (63%)	34.14 (9.06; 20–60)	172.16 (9.66; 153–193)	70.86 (13.69; 47–110)	23.83 (3.79; 18.29–34.33)
Valkinect	35 (51%)	44.69 (11.23; 22–60)	172.60 (8.52; 157–190)	70.91 (11.24; 53–97)	23.76 (3.01; 18.93–32.04)

Data from one randomly selected Valkinect participant with multiple sclerosis was used to exemplify usage of the normative data (male, 53 years, 183 cm, 73 kg).

**Table 2:** Motor task descriptions, information on respectively extracted movement signals and spatiotemporal parameters as well as parameter names. Abbreviations: AP: anterior-posterior; n.u.: unitless; RR: Romberg ratio.

Task Description	Movement Signal and Spatiotemporal Parameter Description	Parameter Names
**Short comfortable speed walk (SCSW)**		
The participant stands just outside the sensor range and walks towards the sensor at comfortable speed in response to an auditory cue	Mean speed derived from pelvic center landmark movement in walk direction	Gait speed [m/s]
	Mean step length, mean step width, and mean step duration over all (left and right) detected steps derived from left and right ankle landmark movement in walk direction	Step length [cm]; Step width [cm]; Step duration [s]
	Mean gait cadence extrapolated from detected steps and recording length	Gait cadence [steps/min]
	Mean angular arm swing amplitude (averaged over left and right averages) and absolute symmetry angle [32] (between left and right mean angular arm swing amplitude) derived from left and right wrist landmarks relative to manubrium landmark movement in anterior-posterior direction	Arm angular amplitude [°]; Arm symmetry angle [n.u.]
**Short maximum speed walk (SMSW)**		
The participant stands just outside the sensor range and walks towards the sensor at maximum speed in response to an auditory cue	Mean speed derived from pelvic center landmark movement in walk direction	Gait speed [m/s]
**Short line walk (SLW)**		
The participant stands just outside the sensor area and, in response to an auditory cue, walks towards the sensor in tandem gait, i.e., walks on an imaginary line with the heels touching the toes at each step	Mean and coefficient of variation of progression speed derived from pelvic center landmark movement in walk direction	Progression speed [°/s]; Relative progression variability [%]
	Angular standard deviation and speed of upper body sway starting from pelvic center landmark	Roll sway variability [°]; Roll sway speed [°/s]
	Line walk cadence derived from recording length and peaks of left and right ankle landmark movement relative to respective hip landmarks	Line walk cadence [steps/min]
	Angular standard deviation and speed of arm movement angle (averaged over left and right) derived from elbow landmarks relative to respective shoulder landmarks movement in 3D	Arm variability [°]; Arm speed [°/s]
**Stepping in place (SIP)**		
The participant walks on the spot at comfortable pace for 40 s	Mean knee amplitude, mean step duration, and mean stance duration (averaged over left and right averages) derived from knee landmark movement in anterior-posterior direction	Knee amplitude [m]; Step duration [s]; Stance duration [s]
	Mean stepping cadence extrapolated from detected steps and recording length	Stepping cadence [steps/min]
	Absolute symmetry angle [32] (between left and right mean knee amplitudes)	Knee symmetry angle [n.u.]
	Mean coefficient of variation of left and right “stride times” measured as time between knee amplitude peaks (i.e., slightly adapted from [23])	Arrhythmicity [%]
**Standing up and sitting down (SAS)**		
The participant sits on an armless chair, arms hanging to the side, stands up after an auditory cue and sits down again after a second auditory cue	Speed of manubrium landmark movement in vertical and anterior-posterior direction	Transition time (up) [s]; Transition time (down) [s]
	Range of manubrium landmark movement in anterior-posterior direction	AP deflection range (up) [m]; AP deflection range (down) [m]
**Postural control (POCO)**		
The participant stands with closed feet and open eyes facing the sensor for 20 s; after an auditory cue subject closes eyes and remains in this position for another 20 s	Angular range and mean speed of the body sway vector between mean ankle landmark position and pelvic center landmark during eyes closed and eyes open measurement conditions in pitch, roll, and 3D direction	Pitch/Roll/3D sway range (open eyes) [°]; Pitch/Roll/3D sway speed (open eyes) [°/s]; Pitch/Roll/3D sway range (closed eyes) [°]; Pitch/Roll/3D sway speed (closed eyes) [°/s]
	Romberg ratio of sway range and sway speed in pitch, roll, and 3D direction—i.e., value for closed eyes condition divided by respective value for open eyes condition	RR of pitch/roll/3D sway range [n.u.]; RR of pitch/roll/3D sway speed [n.u.]

**Table 3:** Descriptive statistics of spatiotemporal parameters and generalizability estimates regarding linear models describing associations with confounders age, sex, height, and weight. Abbreviations: AP: anterior-posterior; CV: cross-validation; CoV: coefficient of variation; n.u.: unitless; Q1: 25th percentile; Q3: 75th percentile; RR: Romberg ratio; SD: standard deviation.

Spatiotemporal Parameter	Mean	SD	CoV	Q1	Q3	Mean $R_{test}^{2}$ for Repeated (100×) 5-Fold CV
**Short comfortable speed walk (SCSW); n = 126**						
Gait speed [m/s]	1.16	0.17	0.15	1.06	1.28	−0.05
Step length [cm]	69.35	7.69	0.11	64.85	74.38	0.14
Step width [cm]	10.19	2.72	0.27	8.24	11.77	0.13
Step duration [s]	0.52	0.05	0.10	0.48	0.56	−0.03
Gait cadence [steps/min]	112.07	10.55	0.09	103.97	120.04	−0.04
Arm angular amplitude [°]	26.48	10.81	0.41	18.19	32.45	−0.14
Arm symmetry angle [n.u.]	0.23	0.16	0.72	0.11	0.30	−0.10
**Short maximum speed walk (SMSW); n = 90**						
Gait speed [m/s]	1.66	0.18	0.11	1.53	1.77	−0.08
**Short line walk (SLW); n = 128**						
Progression speed [m/s]	0.35	0.10	0.28	0.29	0.39	−0.07
Relative progression variability [%]	0.33	0.08	0.24	0.27	0.38	−0.09
Roll sway variability [°]	1.80	0.76	0.42	1.21	2.16	−0.12
Roll sway speed [°/s]	5.58	1.90	0.34	4.37	6.47	−0.14
Line walk cadence [steps/min]	71.78	16.35	0.23	60.50	81.39	−0.07
Arm variability [°]	5.32	3.27	0.62	2.94	6.47	−0.12
Arm speed [°/s]	18.20	7.54	0.41	13.24	20.74	−0.06
**Stepping in place (SIP); n = 121**						
Knee amplitude [m]	0.18	0.06	0.31	0.15	0.23	−0.15
Step duration [s]	0.83	0.11	0.13	0.74	0.88	−0.11
Stance duration [s]	0.39	0.15	0.38	0.28	0.47	0.01
Stepping cadence [steps/min]	98.29	16.13	0.16	87.07	111.00	−0.06
Knee symmetry angle [n.u.]	0.06	0.05	0.87	0.02	0.09	−0.14
Arrhythmicity [%]	6.00	0.84	0.14	5.37	6.50	−0.08
**Standing up and sitting down (SAS); n = 90**						
Transition time (up) [s]	1.53	0.19	0.12	1.39	1.63	−0.16
Transition time (down) [s]	1.66	0.22	0.13	1.48	1.80	−0.09
AP deflection range (up) [m]	0.37	0.07	0.19	0.32	0.41	−0.11
AP deflection range (down) [m]	0.40	0.08	0.20	0.34	0.46	−0.11
**Postural control (POCO); n = 113**						
Pitch sway range (open eyes) [°]	0.91	0.43	0.48	0.59	1.15	−0.13
Roll sway range (open eyes) [°]	0.89	0.37	0.42	0.64	1.09	−0.11
3D sway range (open eyes) [°]	0.92	0.41	0.44	0.67	1.10	−0.14
Pitch sway speed (open eyes) [°/s]	0.14	0.05	0.39	0.10	0.16	−0.10
Roll sway speed (open eyes) [°/s]	0.15	0.06	0.37	0.10	0.18	−0.11
3D sway speed (open eyes) [°/s]	0.22	0.07	0.34	0.17	0.26	−0.10
Pitch sway range (closed eyes) [°]	1.12	0.50	0.45	0.74	1.40	−0.16
Roll sway range (closed eyes) [°]	1.03	0.43	0.41	0.73	1.27	−0.10
3D sway range (closed eyes) [°]	1.09	0.50	0.46	0.71	1.41	−0.15
Pitch sway speed (closed eyes) [°/s]	0.18	0.06	0.36	0.14	0.22	−0.08
Roll sway speed (closed eyes) [°/s]	0.20	0.08	0.39	0.14	0.25	−0.07
3D sway speed (closed eyes) [°/s]	0.30	0.10	0.33	0.21	0.34	−0.06
RR of pitch sway range [n.u.]	1.42	0.75	0.53	0.94	1.75	−0.13
RR of roll sway range [n.u.]	1.29	0.62	0.49	0.84	1.64	−0.13
RR of 3D sway range [n.u.]	1.33	0.69	0.52	0.87	1.65	−0.14
RR of pitch sway speed [n.u.]	1.46	0.62	0.43	1.03	1.77	−0.14
RR of roll sway speed [n.u.]	1.43	0.62	0.43	0.90	1.89	−0.15
RR of 3D sway speed [n.u.]	1.41	0.51	0.36	0.99	1.64	−0.13

**Table 4:** Linear model coefficients describing associations with confounders age, sex, height, and weight. Abbreviations: CI: confidence interval; SCSW: short comfortable speed walk; sϵ: standard deviation of residuals.

Spatiotemporal Parameter	*β*_0_	*β*_0_ *p*-Value; 95% CI	*β_Age_*	*β_Age_ p*-Value; 95% CI	*β_Sex_*	*β_Sex_ p*-Value; 95% CI	*β_Height_*	*β_Height_ p*-Value; 95% CI	*β_Weight_*	*β_Weight_ p*-Value; 95% CI	s_ϵ_
SCSW step length [cm]	−2.176	0.882; [−31.187, 26.835]	−0.073	0.234; [−0.193, 0.048]	−0.913	0.567; [−4.062, 2.236]	0.510	<0.001; [0.331, 0.689]	−0.181	<0.001; [−0.281, −0.080]	6.005
SCSW step width [cm]	6.164	0.274; [−4.953, 17.281]	0.038	0.107; [−0.008, 0.084]	1.301	<0.05; [0.094, 2.507]	−0.020	0.561; [−0.089, 0.048]	0.076	<0.001; [0.038, 0.115]	2.301

## Data Availability

The datasets generated and analyzed as part of this study are available upon reasonable request from the corresponding author for the purpose of replication of results. Participant consent at the time of enrolment did not comprise sharing nor anonymization of individual data.

## Supplementary Materials

The following supporting information can be downloaded at: https://www.mdpi.com/article/10.3390/ijerph192416989/s1, Figure S1. Histograms depicting distributions of age, height, weight, and BMI subdivided by sex. A: Distributions from all studies for motor tasks postural control (POCO), stepping in place (SIP), short comfortable speed walk (SCSW), and short line walk (SLW). B: Distributions from studies Valkinect and VIMS for motor tasks short maximum speed walk (SMSW), standing up and sitting down (SAS); Table S1. Overview of discard rates and exclusion reasons for recordings from studies ASD, Valkinect, and VIMS. Abbreviations: POCO: postural control; rec/s: recording/s; RR: Romberg ratio; SAS: standing up and sitting down; SCSW: short comfortable speed walk; SIP: stepping in place; SLW: short line walk; SMSW: short maximum speed walk; Table S2. Associations of spatiotemporal parameters with factors age, height, weight, sex and study, assessed with Pearson’s correlation coefficients (r), independent samples t-tests or one-way ANOVA respectively. Statistics with respective p-values smaller than 0.05 are highlighted in bold face. Abbreviations: AP: anterior-posterior; n.u.: unitless; RR: Romberg ratio.
