# Supplementary material for: RGB-Depth Camera-Based Assessment of Motor Capacity: Normative Data for Six Standardized Motor Tasks
Source: Int J Environ Res Public Health. 2022 Dec 17;19(24):16989. doi: 10.3390/ijerph192416989 (PMC9779698; doi:10.3390/ijerph192416989)
Supplement: Supplementary file 1 [file ijerph-19-16989-s001.zip › ijerph-1993905-supplementary.pdf]

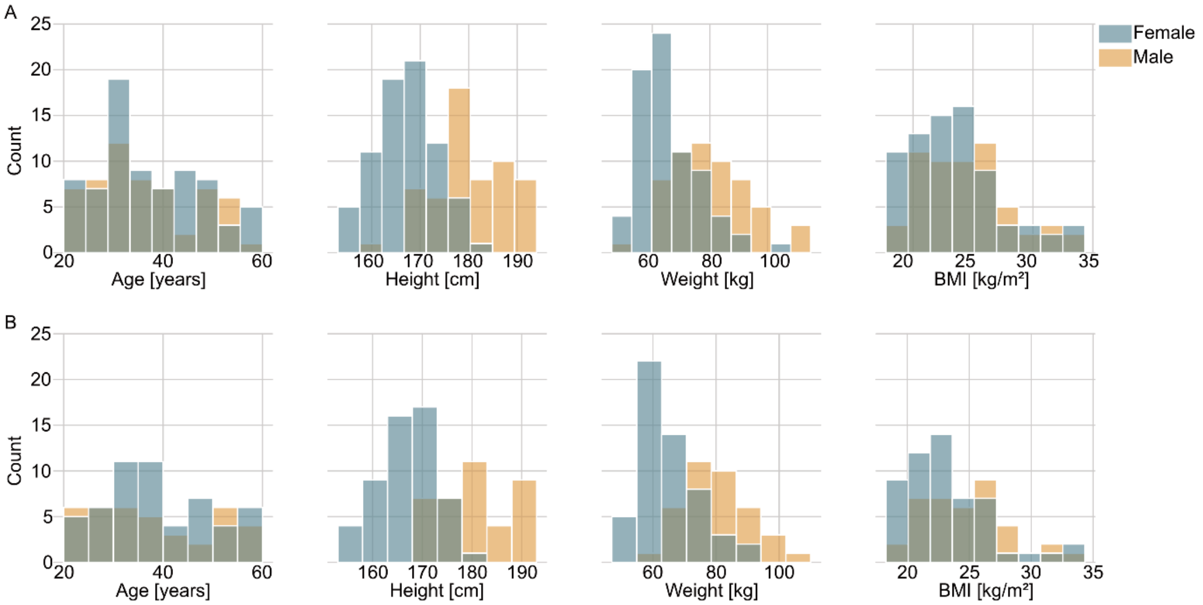

Figure S1. Histograms depicting distributions of age, height, weight, and BMI subdivided by sex. A: Distributions from all studies for motor tasks postural control (POCO), stepping in place (SIP), short comfortable speed walk (SCSW), and short line walk (SLW). B: Distributions from studies Valkinect and VIMS for motor tasks short maximum speed walk (SMSW), standing up and sitting down (SAS).

Table S1. Overview of discard rates and exclusion reasons for recordings from studies ASD, Valkinect, and VIMS. Abbreviations: POCO: postural control; rec/s: recording/s; RR: Romberg ratio; SAS: standing up and sitting down; SCSW: short comfortable speed walk; SIP: stepping in place; SLW: short line walk; SMSW: short maximum speed walk.

**Motor task (studies)**	**Expected number of recs**	**Initial number of recs**	**Discarded**	**Discard Reasons**	**Number of recs after cleaning**
SCSW (ASD, Valkinect, VIMS)	399 recs (3 recs for 133 subjects)	401 recs (3 recs for 131 subjects; 4 recs for 2 subjects)	44 out of 401 (11.0%)	technical issues:<br>- 34 recs: step detection issues, mostly related to unsuitable clothing<br>- 5 recs: arm tracking issues<br>- 1 rec: corrupted depth file<br><br>performance related issues:<br>- 2 recs: rerecorded by operator<br>- 2 recs: unassociated arm movements	357 recs from 126 subjects (3 recs for 1 subject; 2 recs for 15 subjects; 3 recs for 108 subjects).
SMSW (Valkinect, VIMS)	276 recs (3 recs for 92 subjects)	278 recs (3 recs for 90 subjects; 4 recs for 2 subjects)	9 out of 278 (3.2%)	technical issues:<br>- 1 rec: gross signal disturbances in rec environment<br>- 3 recs: lower body not detected<br><br>performance related issues:<br>- 3 recs: subject was jogging<br>- 2 recs: were rerecorded by operator	269 recs from 90 subjects (2 recs for 1 subject; 3 recs from 89 subjects)
SLW (ASD, Valkinect, VIMS)	399 recs (3 recs for 133 subjects)	397 recs (0 recs for 1 subject; 3 recs for 131 subjects; 4 recs for 1 subjects)	32 out of 397 (8.1%)	technical issues:<br>- 1 rec: algorithms failed to extract features<br>- 19 recs: step detection issues (left and right not alternating)<br>- 9 recs: gross signal disturbances, because of unsuitable clothing or parts of body not recorded<br><br>performance related issues:<br>- 1 rec: subject was talking, was repeated by operator<br>- 1 rec: subject walked normally not in tandem gait<br>- 1 rec: subject used wall-support	365 recs from 128 subjects (1 rec for 2 subjects; 2 recs for 15 subjects; 3 recs for 111 subjects)
SIP (ASD, Valkinect, VIMS)	133 recs (1 rec for 133 subjects)	134 recs (1 rec for 132 subjects; 2 recs for 1 subject)	13 out of 134 (9.7%)	technical issues:<br>- 10 recs: step detection issues due to unsuitable clothing, framedrops, or other signal disturbances<br><br>performance related issues:<br>- 2 recs: aborted prematurely, ie duration too short<br>- 1 rec: was rerecorded by operator	121 recs from 121 subjects (1 rec for 121 subjects)
SAS (Valkinect, VIMS)	276 recs (3 recs for 92 subjects)	270 recs (0 recs for 1 subject; 1 rec for 1 subject; 2 recs for 1 subject; 3 recs for 89 subjects)	11 out of 270 (4.1%)	technical issues:<br>- 1 rec: corrupted depth file<br>- 3 recs: implausible between phase transition<br>- 2 recs: only one phase recorded<br>- 2 recs: gross signal disturbance<br><br>performance related issues:<br>- 1 rec: recording stopped prematurely<br>- 2 recs: unassociated movements<br><br>Note: multiple subjects did not have their arms hanging down at the beginning (incorrect according to standard operating procedures) but were not discarded as this was not assumed to affect task performance too severely in healthy controls	259 from 90 subjects (1 rec for 2 subjects; 2 recs for 7 subjects; 3 recs for 81 subjects)
POCO (ASD, Valkinect, VIMS)	133 recs (1 rec for 133 subjects)	133 recs (1 rec for 133 subjects)	20 out of 133 (15.0%)	technical issues:<br>- 5 recs: gross signal disturbance mostly due to unsuitable clothing<br><br>performance related issues:<br>- 14 recs: incorrect feet positioning<br>- 1 rec: sidestep	113 from 113 subjects (1 rec for 113 subjects)

Table S2. Associations of spatiotemporal parameters with factors age, height, weight, sex and study, assessed with Pearson's correlation coefficients (r), independent samples t-tests or one-way ANOVA respectively. Statistics with respective p-values smaller than 0.05 are highlighted in bold face. Abbreviations: AP: anterior-posterior; n.u.: unitless; RR: Romberg ratio.

	**Correlation with Age (*r*; p-value)**	**Correlation with Height (*r*; p-value)**	**Correlation with Weight (*r*; p-value)**	**Sex differences (*t*; p-value)**	**Differences between studies (*F*; p-value)**
**Short comfortable speed walk (SCSW); n=126**					
Gait speed [m/s]	-0.03 (0.77)	0.15 (0.09)	-0.13 (0.15)	0.34 (0.74)	**14.93 (<0.001)**
Step length [cm]	-0.09 (0.32)	**0.43 (<0.001)**	0.01 (0.87)	**3.06 (<0.05)**	**9.75 (<0.001)**
Step width [cm]	0.14 (0.11)	**0.29 (<0.05)**	**0.47 (<0.001)**	**4.27 (<0.001)**	0.28 (0.76)
Step duration [s]	-0.04 (0.69)	**0.29 (<0.001)**	**0.29 (<0.001)**	**2.97 (<0.05)**	**9.89 (<0.001)**
Gait cadence [steps/min]	0.06 (0.51)	**-0.26 (<0.05)**	**-0.28 (<0.05)**	**-2.79 (<0.05)**	**11.39 (<0.001)**
Arm angular amplitude [°]	0.03 (0.74)	-0.01 (0.92)	-0.02 (0.82)	0.05 (0.96)	**14.99 (<0.001)**
Arm symmetry angle [n.u.]	-0.04 (0.63)	-0.14 (0.12)	-0.06 (0.51)	-0.50 (0.62)	**3.33 (<0.05)**
**Short maximum speed walk (SMSW); n=90**					
Gait speed [m/s]	-0.03 (0.79)	**0.26 (<0.05)**	-0.06 (0.55)	**2.56 (<0.05)**	**18.97 (<0.001)**
**Short line walk (SLW); n=128**					
Progression speed [m/s]	0.15 (0.09)	0.04 (0.65)	0.17 (0.06)	**2.18 (<0.05)**	**7.80 (<0.001)**
Relative progression variability [%]	-0.12 (0.18)	0.09 (0.31)	0.17 (0.06)	-0.26 (0.80)	**6.14 (<0.05)**
Roll sway variability [°]	0.09 (0.34)	0.10 (0.27)	**0.20 (<0.05)**	0.22 (0.82)	1.70 (0.19)
Roll sway speed [°/s]	0.09 (0.33)	0.06 (0.50)	**0.25 (<0.05)**	1.04 (0.30)	0.33 (0.72)
Line walk cadence [steps/min]	0.15 (0.09)	**-0.20 (<0.05)**	-0.07 (0.45)	-0.66 (0.51)	**9.47 (<0.001)**
Arm variability [°]	**0.22 (<0.05)**	0.06 (0.53)	0.16 (0.06)	0.24 (0.81)	0.46 (0.63)
Arm speed [°/s]	**0.28 (<0.05)**	-0.04 (0.69)	**0.21 (<0.05)**	0.08 (0.94)	0.49 (0.61)
**Stepping in place (SIP); n=121**					
Knee amplitude [m]	-0.04 (0.63)	0.02 (0.84)	-0.03 (0.73)	0.94 (0.35)	2.97 (0.06)
Step duration [s]	-0.13 (0.16)	0.07 (0.46)	0.12 (0.20)	0.54 (0.59)	**6.28 (<0.05)**
Stance duration [s]	0.07 (0.47)	-0.03 (0.78)	0.02 (0.80)	**2.90 (<0.05)**	0.62 (0.54)
Stepping cadence [steps/min]	0.04 (0.69)	0.00 (1.00)	-0.09 (0.32)	**-2.21 (<0.05)**	**4.89 (<0.05)**
Knee symmetry angle [n.u.]	**0.19 (<0.05)**	0.02 (0.84)	0.04 (0.65)	0.31 (0.75)	0.41 (0.67)
Arrhythmicity [%]	**0.20 (<0.05)**	-0.10 (0.27)	-0.11 (0.23)	-1.95 (0.05)	**5.49 (<0.05)**
**Stand up and sit down (SAS); n=90**					
Transition time (up) [s]	**0.22 (<0.05)**	0.01 (0.92)	0.12 (0.27)	0.27 (0.79)	0.71 (0.40)
Transition time (down) [s]	**0.26 (<0.05)**	0.13 (0.23)	0.19 (0.07)	0.75 (0.46)	**4.59 (<0.05)**
AP deflection range (up) [m]	0.12 (0.25)	0.19 (0.07)	0.06 (0.59)	0.53 (0.60)	0.06 (0.81)
AP deflection range (down) [m]	-0.00 (0.97)	**0.31 (<0.05)**	**0.23 (<0.05)**	1.89 (0.06)	0.02 (0.89)
**Postural control (POCO); n=113**					
Pitch sway range (open eyes) [°]	-0.09 (0.33)	0.11 (0.23)	0.13 (0.18)	0.50 (0.62)	**5.41 (<0.05)**
Roll sway range (open eyes) [°]	-0.03 (0.77)	**0.19 (<0.05)**	0.18 (0.06)	1.04 (0.30)	**3.65 (<0.05)**
3D sway range (open eyes) [°]	-0.07 (0.48)	0.12 (0.20)	0.14 (0.13)	0.79 (0.43)	**4.23 (<0.05)**
Pitch sway speed (open eyes) [°/s]	-0.04 (0.64)	0.13 (0.17)	**0.25 (<0.05)**	0.68 (0.50)	**7.45 (<0.001)**
Roll sway speed (open eyes) [°/s]	-0.01 (0.94)	0.15 (0.11)	**0.26 (<0.05)**	0.44 (0.66)	**7.09 (<0.05)**
3D sway speed (open eyes) [°/s]	-0.03 (0.79)	0.16 (0.09)	**0.27 (<0.05)**	0.63 (0.53)	**9.09 (<0.001)**
Pitch sway range (closed eyes) [°]	0.05 (0.63)	-0.06 (0.53)	0.01 (0.88)	-0.81 (0.42)	0.99 (0.37)
Roll sway range (closed eyes) [°]	-0.01 (0.92)	0.04 (0.66)	0.16 (0.10)	-0.65 (0.52)	0.28 (0.76)
3D sway range (closed eyes) [°]	-0.01 (0.88)	-0.03 (0.75)	0.03 (0.75)	-0.71 (0.48)	0.62 (0.54)
Pitch sway speed (closed eyes) [°/s]	0.10 (0.29)	-0.04 (0.69)	0.15 (0.12)	-1.27 (0.21)	0.95 (0.39)
Roll sway speed (closed eyes) [°/s]	0.04 (0.65)	-0.01 (0.93)	0.15 (0.11)	-1.39 (0.17)	0.98 (0.38)
3D sway speed (closed eyes) [°/s]	0.08 (0.43)	-0.03 (0.76)	0.16 (0.08)	-1.52 (0.13)	1.13 (0.33)
RR of pitch sway range [n.u.]	0.13 (0.18)	-0.16 (0.08)	-0.09 (0.32)	-1.06 (0.29)	**3.74 (<0.05)**
RR of roll sway range [n.u.]	0.03 (0.73)	-0.12 (0.21)	0.05 (0.60)	-0.66 (0.51)	**3.93 (<0.05)**
RR of 3D sway range [n.u.]	0.07 (0.49)	-0.17 (0.07)	-0.08 (0.40)	-1.53 (0.13)	2.41 (0.09)
RR of pitch sway speed [n.u.]	0.13 (0.16)	-0.15 (0.11)	0.01 (0.94)	-1.39 (0.17)	**7.10 (<0.05)**
RR of roll sway speed [n.u.]	0.06 (0.49)	-0.10 (0.29)	-0.00 (0.99)	-0.89 (0.37)	**9.48 (<0.001)**
RR of 3D sway speed [n.u.]	0.09 (0.32)	-0.16 (0.08)	0.01 (0.94)	-1.72 (0.09)	**10.40 (<0.001)**
